# The Effect of Vitamin D Supplementation in Children With Asthma: A Meta-Analysis

**DOI:** 10.3389/fped.2022.840617

**Published:** 2022-06-29

**Authors:** Meiqi Hao, Ruoxin Xu, Nachuan Luo, Miaowen Liu, Junping Xie, Wenxiong Zhang

**Affiliations:** ^1^Department of Thoracic Surgery, The Second Affiliated Hospital of Nanchang University, Nanchang, China; ^2^Jiangxi Medical College, Nanchang University, Nanchang, China; ^3^Department of Respiratory and Critical Care Medicine, The Second Affiliated Hospital of Nanchang University, Nanchang, China

**Keywords:** children, asthma, meta-analysis, vitamin D, systematic review

## Abstract

**Background:**

An increasing number of studies have suggested that vitamin D can be used to treat childhood asthma, but its clinical effects are still unclear. We conducted this meta-analysis to examine the latest estimates of the effectiveness and safety of using vitamin D to treat childhood asthma.

**Methods:**

The PubMed, The Cochrane Library, ScienceDirect, Embase, Scopus, Ovid MEDLINE, Web of Science, and Google Scholar databases were searched for randomized controlled trials (RCTs) describing vitamin D supplementation interventions for asthmatic children. Asthma exacerbation, vitamin D levels, the predicted percentage of forced expiratory volume in the first second (FEV1%) and adverse effects (AEs) were analyzed as the main outcome measures.

**Results:**

After screening, eight RCTs with 738 children were included. Compared with placebos, vitamin D supplementation had a stronger effect on serum vitamin D levels [mean difference (MD) = 13.51 (4.24, 22.79), *p* = 0.004]. The pooled results indicated that no significant changes were found between the groups in asthma control, as measured by adopting the following indicators: asthma exacerbation [risk ratio (RR) = 0.92 (0.68, 1.25), *p* = 0.60]; Childhood Asthma Control Test (CACT) scores [MD = 0.15 (−0.43, 0.74), *p* = 0.61]; hospitalizations for asthma exacerbation [RR = 1.20 (0.48, 2.96), *p* = 0.70]; acute care visits [RR = 1.13 (0.77, 1.65), *p* = 0.63]; steroid use [RR = 1.03 (0.41, 2.57), *p* = 0.95]; and fractional exhaled nitric oxide (FeNO) [MD =-3.95 (−22.87, 14.97), *p* = 0.68]. However, vitamin D supplementation might reduce the FEV1% [MD = −4.77 (−9.35, −0.19), *p* = 0.04] and the percentage of predicted forced vital capacity (FVC%) [MD =-5.01 (−9.99, −0.02), *p* = 0.05] in patients. Subgroup analysis revealed no difference in AEs between the two groups.

**Conclusions:**

Vitamin D supplementation significantly increased patients' serum vitamin D levels, but it had no benefit for asthma control. However, vitamin D supplementation might reduce patients' lung function. It is essential to systemically search for more large-scale, rigorous, and well-designed RCTs to fully confirm these conclusions.

**Systematic Review Registration:**

https://www.crd.york.ac.uk/prospero/display_record.php?ID=CRD42021288838, PROSPERO CRD42021288838.

## Introduction

Asthma is a chronic inflammation of the airway involving a variety of inflammatory cells. Children account for a large proportion of asthma patients, and it is considered a significant global health burden (1–3). The treatment strategy follows the recommendation of the Global Asthma Initiative (GINA) for childhood asthma, and the key is to control airway inflammation (4).

Vitamin D has potential prospects in the treatment of childhood asthma. Vitamin D is considered to be a complex immunomodulatory molecule, and its role in anti-inflammatory effects and regulating the immune response has attracted increasing attention (5, 6).

The conclusions regarding the efficacy of vitamin D for treating childhood asthma have been inconsistent. The traditional prevention or management methods include long-acting β2 receptor agonists, oral steroids and inhaled corticosteroids (ICS) (7). Although the regular use of ICS and other treatments can reduce mortality, the prevalence and incidence of the disease are still increasing (8). Recent studies have shown that there is a close link between a high asthmatic incidence and vitamin D deficiency (9–11). Increasing evidence has shown that vitamin D is a safe, effective and cost-effective therapy for asthma (12). Some trials have demonstrated that vitamin D can help improve lung function, enhance asthma control and reduce disease severity in the field of asthma therapy (13–15). However, not all studies that have examined vitamin D have reported favorable effects, and there are still disagreements about its effects. Some researchers have also found that vitamin D does not improve asthma control and may even have a negative effect on lung function (16, 17).

To resolve this inconsistency and explore the safety of vitamin D for asthmatic children, we conducted this study by analyzing the relevant literature.

## Materials and Methods

Our study was conducted in accordance with the Preferred Reporting Items for Systematic Review and Meta-Analysis (PRISMA) statement, and more details can be found in Supplementary Table S1. (Registration information: PROSPERO CRD42021288838).

### Search Strategy

We comprehensively searched the PubMed, The Cochrane Library, ScienceDirect, Embase, Scopus, Ovid MEDLINE, Google Scholar and Web of Science databases for eligible RCTs from inception to 24 October 2021. “Asthma,” “vitamin D,” and “children” were used as keywords. We also manually searched the references lists of the included RCTs for additional eligible studies (Supplementary Table S2).

### Selection Criteria

Two researchers independently screened relevant articles using Endnote. The inclusion criteria were as follows: (1) population: children (up to 18 years of age) with asthma (doctor's diagnosis and/or objective criteria); (2) intervention and control: vitamin D vs. a placebo; (3) outcomes: CACT scores (18), asthma exacerbation, hospitalizations for asthma exacerbation, steroid use, FeNO, acute care visits, FEV1%, FVC%, FEV1: FVC ratio, serious adverse events, and vitamin D levels; and (4) study design: RCT.

### Data Extraction

Two investigators independently screened the information and compiled it into a table. The third researcher had no disagreements in this process. The following data were extracted: name of the first author, year of publication, study country, participants' basic characteristics (age, intervention, number, baseline data), asthma control parameters (CACT scores, asthma exacerbation, hospitalizations for asthma exacerbation, acute care visits, steroid use and FeNO), lung function parameters (FEV1%, FVC%, the FEV1: FVC ratio), safety (AEs), and vitamin D levels.

### Quality Assessment

The 5-point Jadad scale (19) was applied to evaluate the quality of the RCTs. The scale includes three questions about randomization, masking and the accountability of the trial. Each question was scored, and if the sum of the final score was more than three points, the article was considered high-quality.

### Statistical Analysis

The final statistical analysis of the data was performed using Review Manager 5.4 software and STATA 15.0 software. The MD and 95% confidence intervals (CIs) were used to assess FEV1%, FEV1%, the FEV1: FVC ratio, CACT scores, vitamin D levels (if the MD was > 0, the factor was considered beneficial to the vitamin D group, while an MD < 0 indicated that the factor indicated that the factor was beneficial to the placebo group), and FeNO (if the MD was < 0, the factor was considered beneficial to the vitamin D group, while an MD > 0 indicated that the factor indicated that the factor was beneficial to the placebo group). RRs with 95% CIs were used to analyze asthma exacerbation, hospitalizations for asthma exacerbation, adverse events, acute care visits, and steroid use (if the RR was < 1, the factor was considered beneficial to the vitamin D group, while an RR > 1 indicated that the factor was beneficial to the placebo group). If *I*^2^ > 50%, the results were considered to have heterogeneity, and the random effects model was employed; when *I*^2^ was <50%, the results were considered to have no low heterogeneity, and a fixed effects model was employed. We also employed Egger's test and Begg's test to quantify the funnel chart and obtain a *p* value to determine whether publication bias was present. When the *P* value was lower than 0.05, it was considered statistically significant.

## Results

### Search Results and Study Quality Assessment

**Figure 1:**
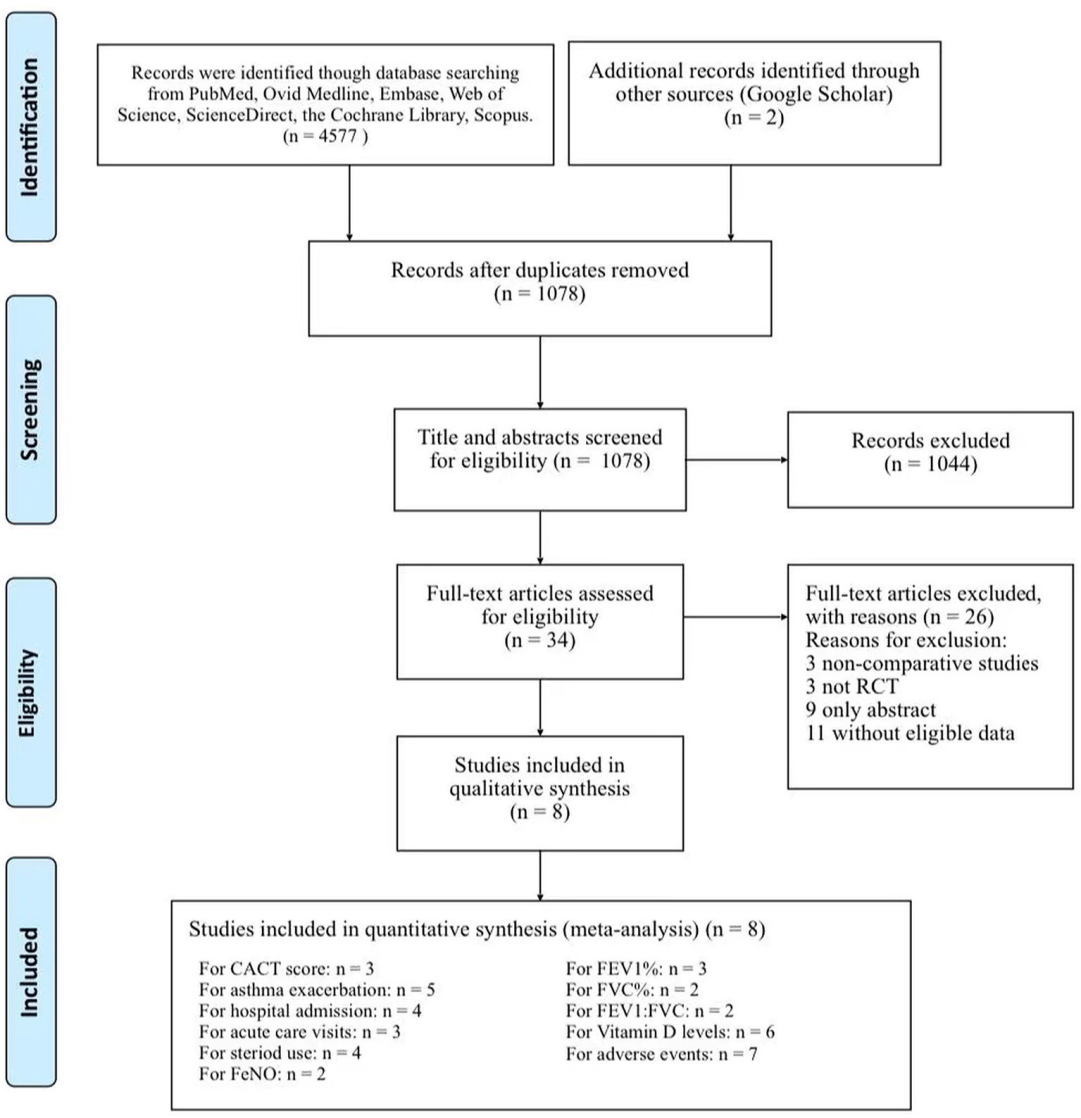
Flow chart of study selection.

The initial searched yielded 4,577 articles. A total of 1,078 duplicate studies were deleted. After title and abstract screening, 34 potentially relevant studies remained. After reading the full texts of these studies, eight RCTs involving 738 children (376 children took vitamin D and 362 took placebos) were included in our meta-analysis (Figure 1) (16, 17, 20–25). Among the 8 RCTs, seven studies were considered to be high-quality [six had scores of 5 points (17, 21–25) and one had a score of 4 points (20) on the Jadad scale], and one study (16) was considered to be medium-quality (3 points on the Jadad scale) (Supplementary Table S3). The geographical distribution of studies was relatively wide, with four studies in Asia (17, 20, 21, 25) and four in North America (16, 22–24). More details on the included RCTs, including baseline characteristics and main outcome indicators, are shown in Table 1.

**Table 1:** Summary of the baseline characteristics of the included studies.

**Study**		**Country**	**Period (year)**	**Groups**	**Patients**	**Sex** **(M/F)**	**Age (Mean, year)**	**Duration**	**Oral dose**	**Baseline characteristics**					**Quality (score)**	**Follow up**
										**Vitamin D levels**	**CACT** **(score)**	**Lung fuction (%)**				
											**FEV1%**	**FVC%**	**FEV1:FVC**			
2021	Thakur (25)	India	2018.07– 2019.11	Vitamin D	30	16/14	9.0	12 weeks	2,000 IU/day	15.8 ±8.2	18.0 ± 2.9	75.3 ± 26.5	NA	NA	5	3 months
				Placebo	30	18/12	8.7			16.5 ± 9.9	15.5 ± 2.7	75.6 ± 15.7				
2021	Jat (17)	India	2017.05– 2019.08	Vitamin D	125	89/36	8.2	9 months	1,000 IU/day	11.6 ± 4.6	21.7 ± 4.2	92.5 ± 21.7	92.7 ± 21.7	98.5 ± 10.9	5	9 months
				Placebo	125	91/34	7.8			10.8 ± 4.4	21.9 ± 3.6	97.0 ± 17.5	94.6 ± 17	99.3 ± 10.1		
2020	Forno (24)	USA	2016.02– 2019.03	Vitamin D	96	52/44	9.9	48 weeks	4,000 IU/day	22.5 ± 4.6	22.0 ± 3.2	93.9 ± 15.8	NA	91.5 ± 9.3	5	48 weeks
				Placebo	96	63/33	9.7			22.8 ± 4.6	21.3 ± 3.6	90.6 ± 17.3		89.6 ± 10.1		
2019	Ducharme (23)	Canada	2014.09– 2015.11	Vitamin D	23	16/7	2.9	0, 3.5 months	100,000 IU at baseline and 3.5 months	28.5 ± 5.8	NA	NA	NA	NA	5	7 months
				Placebo	24	14/10	2.9			29.4 ± 11.1						
2016	Tachimoto (21)	Japan	2010.10– 2013.04	Vitamin D	54	28/26	10.0	2 months	800 IU/day	28.1 ± 7.6	25.0 ± 3.0	87.2 ± 6.1	98.1 ± 11.4	87.6 ± 5.3	5	6 months
				Placebo	35	22/13	9.8			29.7 ± 7.7	26.0 ± 1.5	87.1 ± 5.4	96.6 ± 11.6	86.4 ± 7.0		
2016	Kerley (16)	Ireland	2013.11– 2014.04	Vitamin D	17	11/6	10	15 weeks	2,000 IU/day	20.45 ± 7.43	19.0 ± 3.2	105.0 ± 16.3	94.1 ± 11.3	94.2 ± 8.9	3	15 weeks
				Placebo	22	13/9	7			20.45 ± 8.92	16.7 ± 3.7	96.0 ± 10.37	91.6 ± 9.5	93.3 ± 6.3		
2016	Jenson (22)	Canada	2013.11– 2014.08	Vitamin D	11	4/7	2.2	6 months	100 000 IU bolus then 400 IU/day	24.8 ± 2.5	NA	NA	NA	NA	5	6 months
				Placebo	11	3/8	3.1		400 IU vitamin D / day	27.2 ± 2.5						
2015	Bar (20)	Israel	unclear	Vitamin D	20	12/8	13.5	6 weeks	14,000 IU/week	20.8 ± 6.5	NA	NA	NA	NA	4	6 weeks
				Placebo	18	13/6	12.4			20.0 ± 7.1						

*CACT, childhood asthma control test; Data are mean (±SD); FEV1%, predicted percentage of forced expiratory volume in first second; FVC%, percentage of predicted forced vital capacity; IU, international unit; M/F, male/female; NA, not available*.

Two reviewers assessed the quality of the 8 studies based on the Cochrane Handbook (26). The handbook clarified that the risk of bias in RCTs needs to be assessed by examining following criteria: random sequence generation, allocation concealment, blinding of the participants and personnel, blinding of the outcome assessment, incomplete outcome data, selective reporting, and other bias (Supplementary Figure S1). Six studies (17, 21–25) described random sequence generation and were regarded as having a low risk of bias, but two studies (16, 20) had an unclear risk of bias because of a lack of description. Seven studies (17, 20–25) described the allocation concealment process and were regarded as having a low risk of bias, and only one study (16) was considered to have an unclear risk of bias. In the domain of blinding of participants and personnel and outcome assessment, all studies described blinding assignments, researchers, and patients. Thus, all the studies were regarded as having a low risk of bias. For incomplete outcome data, two (21, 23) had an unclear risk of bias because of the lack of outcome data. For the domain of selective reporting and other biases, all the studies were regarding as having a low risk of bias. Disagreements between the two reviewers were resolved *via* discussion or by consulting a third researcher.

In addition, to evaluate the quality of evidence for each terminal, we adopted the Grading of Recommendations Assessment, Development, and Evaluation (GRADE) system (27). This system divided the quality of evidence into four levels by evaluating the risk of bias, inconsistency, indirectness, imprecision, and publication bias. Supplementary Table S4 summarizes the statistical results and the quality of evidence. We assessed the quality of all outcome measures, and the associated qualities of evidence were rated as very low or low. Consequently, the results should be interpreted cautiously.

### Vitamin D Levels

**Figure 2:**
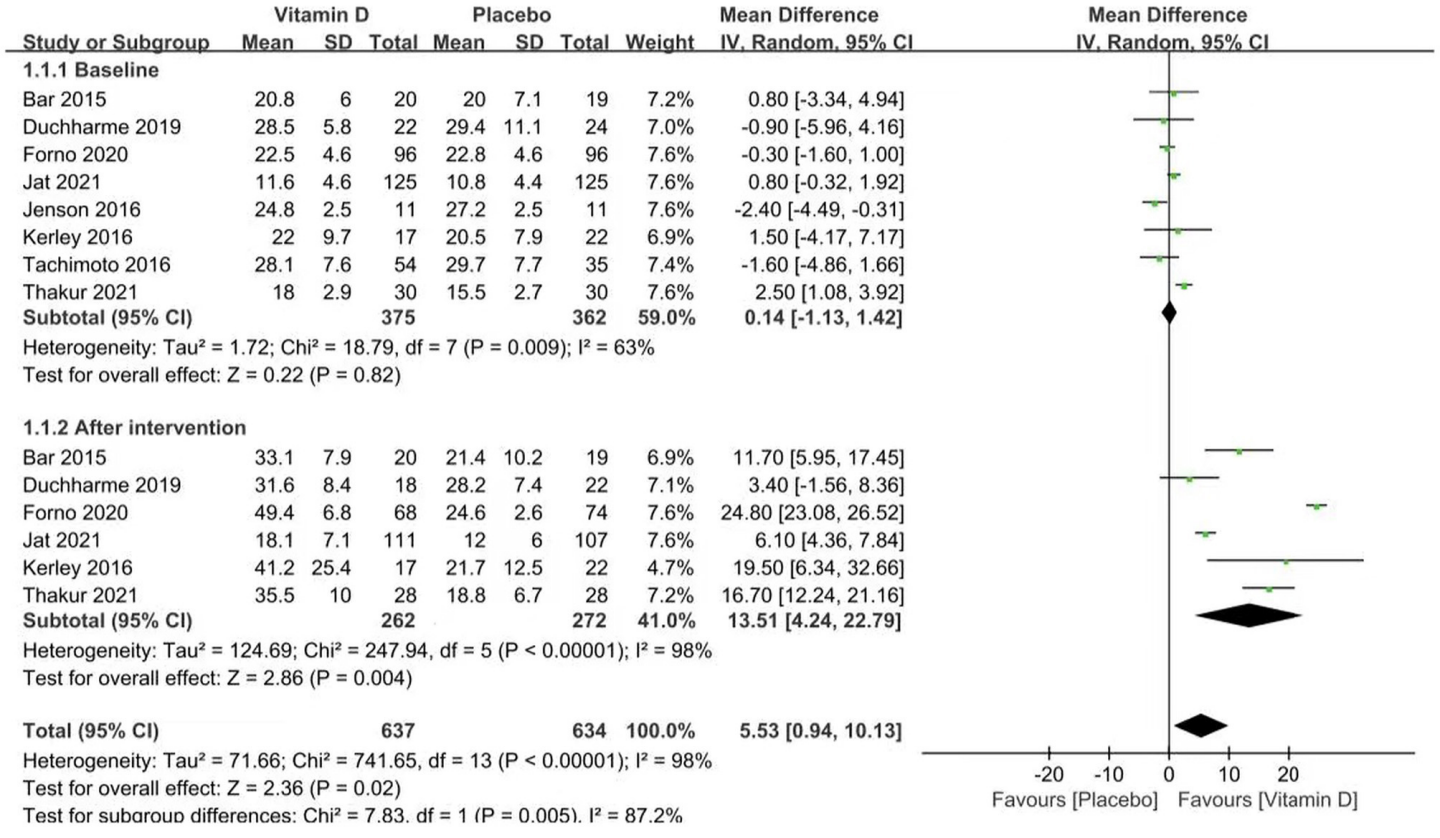
Forest plots of MD of vitamin D levels associated with vitamin D vs. placebo.

Six studies reported vitamin D levels (heterogeneity: *p* < 0.00001, *I*^2^ = 98%). The vitamin D levels of both groups improved compared with the baseline, and the vitamin D group had a stronger effect [MD = 13.51 (4.24, 22.79), *p* = 0.004; Figure 2].

### Asthma Control

We evaluated asthma control between the two groups by examining CACT scores (18), FeNO, asthma exacerbation, hospitalizations for asthma exacerbation, acute care visits and steroid use.

The included studies did not provide a clear definition of asthma exacerbation. Therefore, variable definitions reported in major publications were utilized in our meta-analysis. It is defined as increased symptoms of shortness of breath, cough, wheezing or chest tightness, and a progressive decline in lung function or the need for a change in treatment.

**Figure 3:**
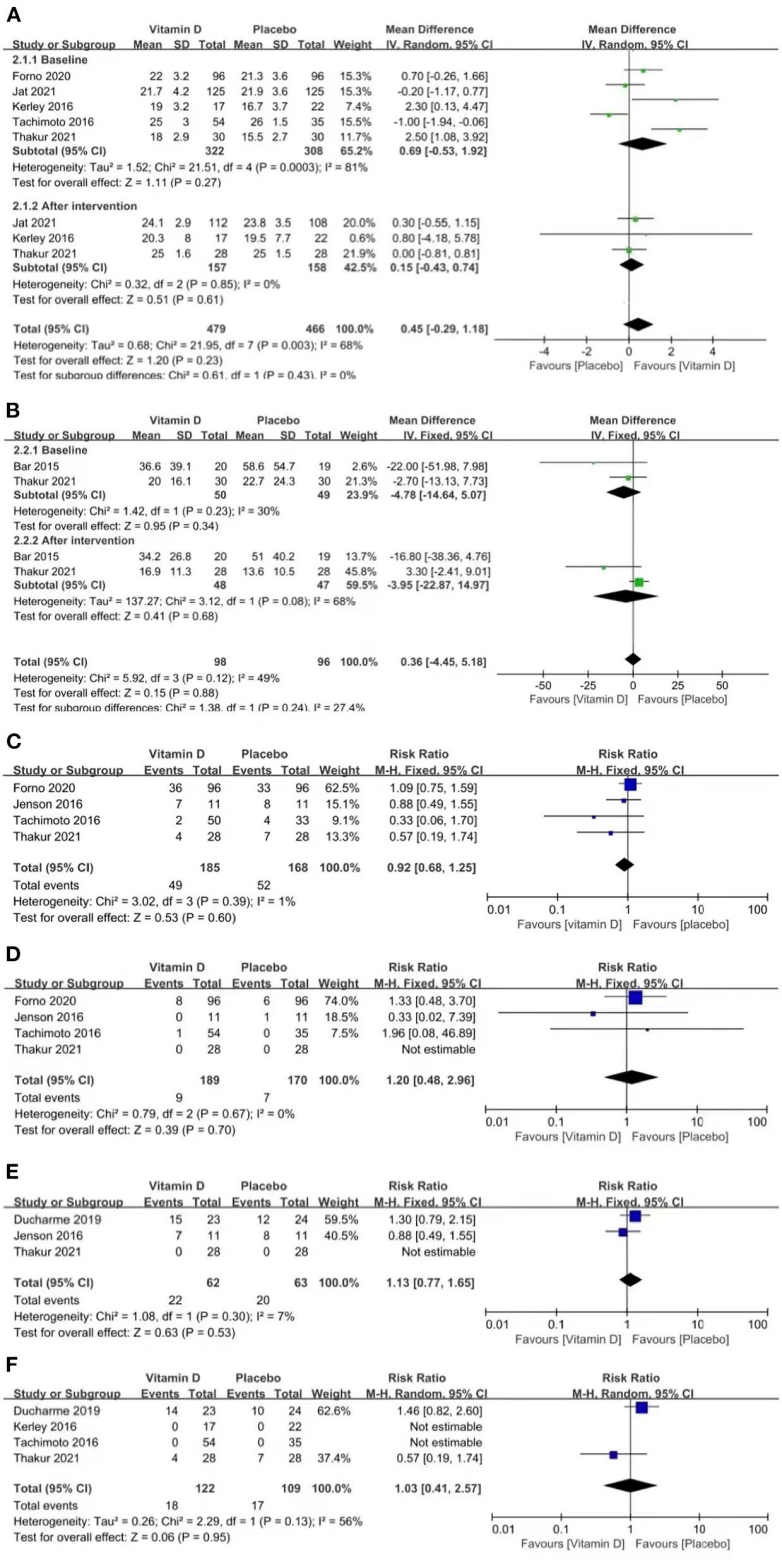
Forest plots of MD of CACT scores **(A)** and FeNO **(B)**, and RR of asthma exacerbation **(C)**, hospitalizations for asthma exacerbation **(D)**, acute care visits **(E)**, and steroid use **(F)** associated with vitamin D vs. placebo.

Three studies reported CACT scores (*p* = 0.85, *I*^2^ = 0%). The CACT scores improved in both groups compared to the baseline data, but no significant difference was found between the groups [MD = 0.15 (−0.43, 0.74), *p* = 0.61; Figure 3A].

Two studies reported FeNO (*p* = 0.08, *I*^2^ = 68%). FeNO was reduced in both groups compared to the baseline data, but no significant difference was found between the groups [MD = −3.95 (−22.87, 14.97), *p* = 0.68; Figure 3B].

Four studies reported asthma exacerbation (*p* = 0.39, *I*^2^ = 1%). The pooled results indicated that the results between the two groups were similar, which suggests that vitamin D is not capable of improving asthma exacerbation [RR = 0.92 (0.68, 1.25), *p* = 0.60; Figure 3C].

Four studies reported hospitalizations for asthma exacerbation (*p* = 0.67, *I*^2^ = 0%). The pooled results indicated that vitamin D did not improve the rate of hospitalizations for asthma exacerbation [RR = 1.20 (0.48, 2.96), *p* = 0.70; Figure 3D].

Three studies reported acute care visits (*p* = 0.30, *I*^2^ = 7%). The pooled results indicated that vitamin D did not improve the rate of acute care visits [RR = 1.13 (0.77, 1.65), *p* = 0.53; Figure 3E].

Four studies reported steroid use (*p* = 0.13, *I*^2^ = 56%). The pooled results indicated that vitamin D did not improve the rate of steroid use [RR = 1.03 (0.41, 2.57), *p* = 0.95; Figure 3F].

### Lung Function

We evaluated lung function by examining FEV1%, FVC%, and the FEV1: FVC ratio between the two groups.

FEV1%, FVC% and the FEV1: FVC ratio are all indicators of ventilation function, which decreases when asthma attacks occur. FEV1% < 80% and FEV1: FVC% < 70% are extremely important indicators of airflow restriction. These coincident indicators may gradually recover during remission.

**Figure 4:**
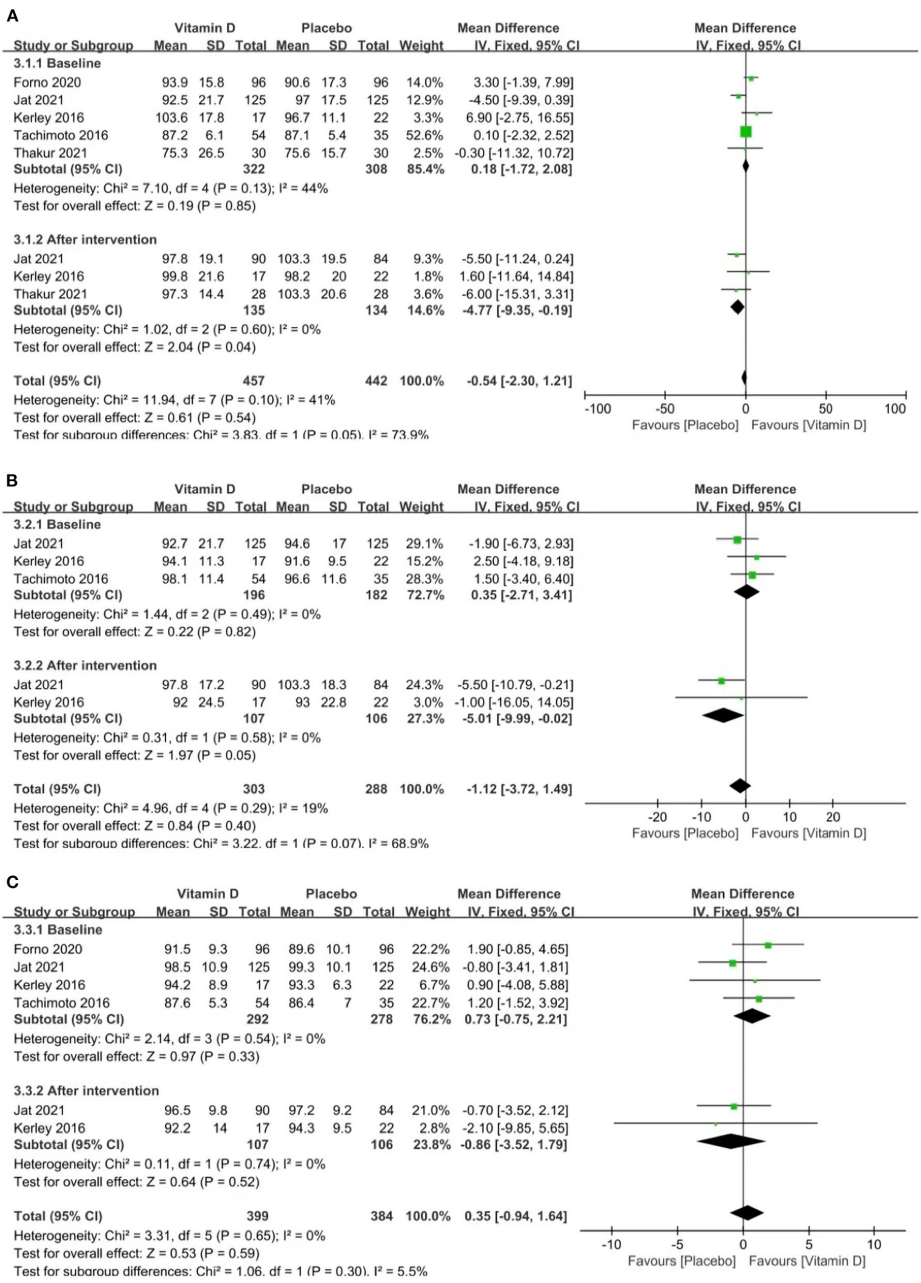
Forest plots MD of FEV1% **(A)**, FVC% **(B)**, and FEV1: FVC **(C)** associated with vitamin D vs. placebo.

Three studies reported FEV1% (heterogeneity: *p* = 0.60, *I*^2^ = 0%). The pooled results showed that the influence of placebos on FEV1% was stronger than that of vitamin D [MD = −4.77 (−9.35, −0.19), *p* = 0.04; Figure 4A].

Two studies reported FVC% (*p* = 0.58, *I*^2^ = 0%). The pooled results showed that the influence of placebos on FVC% was stronger than that of vitamin D [MD = −5.01 (−9.99, −0.02), *p* = 0.05; Figure 4B].

Two studies reported the FEV1: FVC ratio (*p* = 0.74, *I*^2^ = 0%). The pooled results indicated no relevance between vitamin D supplementation and the FEV1: FVC ratio [MD = −0.86 (−3.52, 1.79), *p* = 0.52; Figure 4C].

### Safety

We evaluated safety in the form of serious adverse events between the two groups.

Four studies reported serious adverse events (*p* = 0.55, *I*^2^ = 0%). The analysis results of the two groups were similar [RR = −0.86 (−3.52, 1.79), *p* = 0.99; Supplementary Figure S2].

We conducted a subgroup analysis of AEs and compiled them into a table (Table 2). The summary results indicated that no significant difference was found in AEs.

**Table 2:** Adverse effects (all grade) associated with vitamin D vs. placebo.

**Adverse effects**	**Vitamin D group**	**Placebo group**	**RR (95% CI)**	***P* value**	**Heterogeneity**	
	**(event/total)**	**(event/total)**				
					***I^**2**^* (%)**	***P* value**
Hypercalciuria	5/62	9/63	0.58 [0.22, 1.53]	0.27	0	0.65
Blood and lymphatic system disorders	5/119	1/120	3.76 [0.64, 22.11]	0.14	0	0.80
Infection	19/23	17/24	1.17 [0.85, 1.60]	0.34	Not applicable	
General disorders	13/23	17/24	0.80 [0.51, 1.24]	0.32	Not applicable	
Investigations	7/23	9/24	0.81 [0.36, 1.82]	0.61	Not applicable	
Nausea	7/249	8/249	0.82 [0.53, 1.27]	0.38	Not applicable	
Constipation	11/249	12/249	0.92 [0.42, 2.00]	0.83	Not applicable	
Vomiting	28/249	34/249	0.82 [0.53, 1.27]	0.38	Not applicable	
Pain abdomen	41/249	40/249	1.02 [0.72, 1.47]	0.89	Not applicable	
Headache	25/153	25/153	1.00 [0.61, 1.64]	1.00	Not applicable	
Ear and labyrinth disorders	0/23	2/24	0.21 [0.01, 4.12]	0.30	Not applicable	
Eye disorders	0/23	1/24	0.35 [0.01, 8.11]	0.51	Not applicable	
Gastrointestinal disorders	3/23	8/24	0.39 [0.12, 1.30]	0.12	Not applicable	
Immune system disorders	2/23	5/24	0.42 [0.09, 1.94]	0.27	Not applicable	
Musculoskeletal disorders	0/23	1/24	0.35 [0.01, 8.11]	0.51	Not applicable	
Nervous system disorders	1/23	1/24	1.04 [0.07, 15.72]	0.98	Not applicable	
Altered sensorium	1/125	0/125	3.00 [0.12, 72.94]	0.50	Not applicable	
Seizures	0/125	1/125	0.33 [0.01, 8.10]	0.50	Not applicable	
Reproductive system and breast disorders	1/23	0/24	3.13 [0.13, 73.01]	0.48	Not applicable	
Respiratory, thoracic and mediastinal disorders	4/23	7/24	0.60 [0.20, 1.77]	0.35	Not applicable	
Skin and subcutaneous tissue disorders	7/23	4/24	1.83 [0.62, 5.42]	0.28	Not applicable	
Surgical and medical procedure	1/23	1/24	1.04 [0.07, 15.72]	0.98	Not applicable	

*CI, confidence interval; RR, Risk Ratio*.

### Subgroup Analysis

To determine whether the duration of vitamin D supplementation affected the results, we performed a subgroup analysis of CACT scores (Supplementary Figure S3A), asthma exacerbation (Supplementary Figure S3B) and FEV1% (Supplementary Figure S3C) by dividing the duration into <6 months and >6 months. Subgroup analysis revealed no significant differences in CACT scores, FEV1% or asthma exacerbation between the vitamin D and placebo groups.

### Sensitivity Analysis

The pooled results showed that vitamin D levels had high heterogeneity (*I*^2^ > 50%). Therefore, we conducted a sensitivity analysis of vitamin D levels and FEV1%, which is the lung function index. To explore the stability and sensitivity of the outcomes, we assessed the influence of each study on the aggregated results, which indicated that the results regarding vitamin D levels (Supplementary Figure S4A) and FEV1% (Supplementary Figure S4B) were stable and reliable.

### Publication Bias

Latent publication bias was not found in vitamin D levels (Egger's test: *p* = 0.408; Begg's test: *p* = 0.260) and FEV1% (Egger's test: *p* = 0.576; Begg's test: *p* = 0.296). The specific analysis is shown in Supplementary Figures S5A,S5B.

## Discussion

In recent years, the global prevalence of asthma in children has been increasing, and this fact has increasingly attracted the attention of researchers. (1, 2, 28). Whether vitamin D can be employed clinically to control childhood asthma remains to be verified due to inconsistent findings (12–17). Through the analysis of 8 high-quality RCTs (16, 17, 20–25), we directly compared the influences of vitamin D and placebos on the treatment for childhood asthma, and this is the first meta-analysis based on RCTs exploring the effects and safety of vitamin D supplementation on childhood asthma. Our meta-analysis provides the latest clinical evidence that vitamin D supplementation can significantly enhance serum vitamin D levels, which fails to improve asthma control for pediatric patients. However, vitamin D supplementation may reduce patients' lung function. Regarding vitamin D toxicity, the pooled outcomes showed similar results between the two groups in the form of AEs.

Compared with the placebos, vitamin D affected serum vitamin D levels in a stronger way. However, almost all the included outcome measures did not show favorable changes in asthma control. Four studies proposed definitions of asthma exacerbation (23, 24, 26, 27). The CACT is a validated scale (18) that includes seven questions, and scores on the scale range from 0 to 27. A score > 19 indicates good symptom control, and higher scores indicate better symptom control. Elevated FeNO levels are found in atopic and allergic patients and are associated with airway hyper reactivity and sputum eosinophils (29). This suggests that if vitamin D supplementation does reduce inflammation, FeNO levels in the vitamin D group would be expected to decrease. However, it was observed that there were no changes between the groups in the aspect of CACT scores and FeNO levels. Among the included studies, only one showed favorable results on asthma control after the intervention (23). In contrast, a study in India (30) also found that vitamin D intervention could shorten the severity of asthma attacks for children. The curative effect of vitamin D in improving lung function as well as controlling asthma remains highly controversial. The disagreement may be due to the limited effectiveness of the intervention for children with sufficient vitamin D levels. In many current studies, clinical heterogeneity, small sample sizes or other inaccurate factors still exist, which can all lead to inaccurate analysis results.

Our meta-analysis results indicated that vitamin D intervention has a certain adverse effect on lung function, although many observational studies have described the beneficial aspects of vitamin D interventions on lung function (13–15). Contrary to our conclusion, both groups showed improvement in FEV1 and FVC% after vitamin D supplementation, but the influence on the placebo group was more stronger than that on the vitamin D group. We further analyzed the baseline data of FEV1 and FVC% from both groups, and there was no significant change between them. This may be due to high vitamin D levels having a negative impact on lung function. Some studies have also found that vitamin D interventions had no other advantageous changes (16, 31). One possible explanation for the adverse effect of vitamin D on asthmatic children is their high baseline levels, which may leave little room for improvements in lung function. As the latent adverse effects of vitamin D on children's lung function remain to be further explored, physicians must be cautious in the use of vitamin D for asthmatic children.

Vitamin D toxicity remains a problem and is characterized by severe hypercalcemia (32). In our analysis, we found that the rates of AEs between the two groups of children taking vitamin D and placebos were not significantly different. The data from the pooled results verified the safety of vitamin D at therapeutic doses for asthmatic children. This conclusion was consistent with the results reported by other studies (17, 27).

We recognize that there were still many limitations to this analysis. First, we only included eight RCTS, and the limited sample size of pediatric patients included in the analysis may affect the quality of the outcomes. Second, although the outcomes of the analysis showed the number and severity of asthma exacerbations were similar between the two groups, there were discrepancies in the definition of asthma exacerbation in the included trials, which also made the results less reliable. Third, the doses and durations of intervention in the studies were not the same. The optimal duration and dose of vitamin D needed to effectively control asthma symptoms are still unclear. Fourth, the phenotypes of asthma included in the study varied, which also might lead to study heterogeneity and weaken the quality of the results. It must be pointed out that the influence of vitamin D on asthma control may also be affected by genetic differences in vitamin D metabolic pathways (33). However, the influence of genetic factors was not evaluated in our meta-analysis. Because of the above deficiencies, additional larger, rigorously designed RCTs are still needed to further explore the possible benefits of vitamin D for childhood asthma therapy.

In conclusion, vitamin D supplementation affected the serum vitamin D levels of asthmatic children significantly, but failed to improve their asthma control. Vitamin D supplementation might even reduce patients' lung function. The rate of AEs was similar between the children taking vitamin D and those taking placebos, so it was generally believed that taking vitamin D was safe. Due to the limitations of the study, it is essential to perform more large-scale, rigorous and well-designed RCTs to fully confirm this conclusion.

## Data Availability Statement

The original contributions presented in the study are included in the article/Supplementary Material, further inquiries can be directed to the corresponding author.

## Author Contributions

Full access to all of the data in the manuscript, takes responsibility for the integrity of the data, and the accuracy of the data analysis: MH and WZ. Drafting of the manuscript: MH, RX, NL, and ML. Critical revision of the manuscript for important intellectual content: MH and WZ. Statistical analysis and supervision: MH, JX, and WZ. Concept and design, acquisition, analysis, or interpretation of data: All authors. All authors contributed to the article and approved the submitted version.

## Funding

This study was supported by National Natural Science Foundation of China (NSFC, Grant Nos: 81160294 and 81960425), Natural Science Foundation of Jiangxi Province (Grant No: 20212BAB206050), Science and technology planning project of Health Commision of Jiangxi Province (Grant No: 202110045), and Science and technology planning project of Jiangxi Administration of traditional Chinese Medicine (Grant No: 2020B0108). Role of the Funding: The funding had no role in the design and conduct of the study; collection, management, analysis, and interpretation of the data; preparation, review, or approval of the manuscript, and decision to submit the manuscript for publication.

## Conflict of Interest

The authors declare that the research was conducted in the absence of any commercial or financial relationships that could be construed as a potential conflict of interest.

## Publisher's Note

All claims expressed in this article are solely those of the authors and do not necessarily represent those of their affiliated organizations, or those of the publisher, the editors and the reviewers. Any product that may be evaluated in this article, or claim that may be made by its manufacturer, is not guaranteed or endorsed by the publisher.

## Supplementary Material

Supplementary Figure S1 Risk of bias graph for included studies.

Supplementary Figure S2 Forest plots RR of serious adverse events associated with vitamin D versus placebo.

Supplementary Figure S3 Subgroup analysis of CACT scores, asthma exacerbation and FEV1%.

Supplementary Figure S4 Meta-based influence analyses for vitamin D levels **(A)** and FEV1% **(B)**.

Supplementary Figure S5 Egger's and Begg's tests for comparisons of vitamin D levels **(A)** and FEV1% **(B)**.

## Abbreviations

AEs: adverse effects
CACT: childhood asthma control test
CI: confidence interval
FeNO: fractional exhaled nitric oxide
FEV1%: predicted percentage of forced expiratory volume in first second
FVC%: percentage of predicted forced vital capacity
GINA: the Global Initiative for Asthma
GRADE: The Grading of Recommendations Assessment, Development and Evaluation
ICS: include inhaled corticosteroids
MD: Mean Difference
PRISMA: Preferred Reporting Items for Systematic Review and Meta-Analysis
RCT: randomized controlled trial
RR: Risk Ratio.
