# Supplementary figures and images for: The Effect of Vitamin D Supplementation in Children With Asthma: A Meta-Analysis
Source: Front Pediatr. 2022 Jun 29;10:840617. doi: 10.3389/fped.2022.840617 (PMC9277022; doi:10.3389/fped.2022.840617)

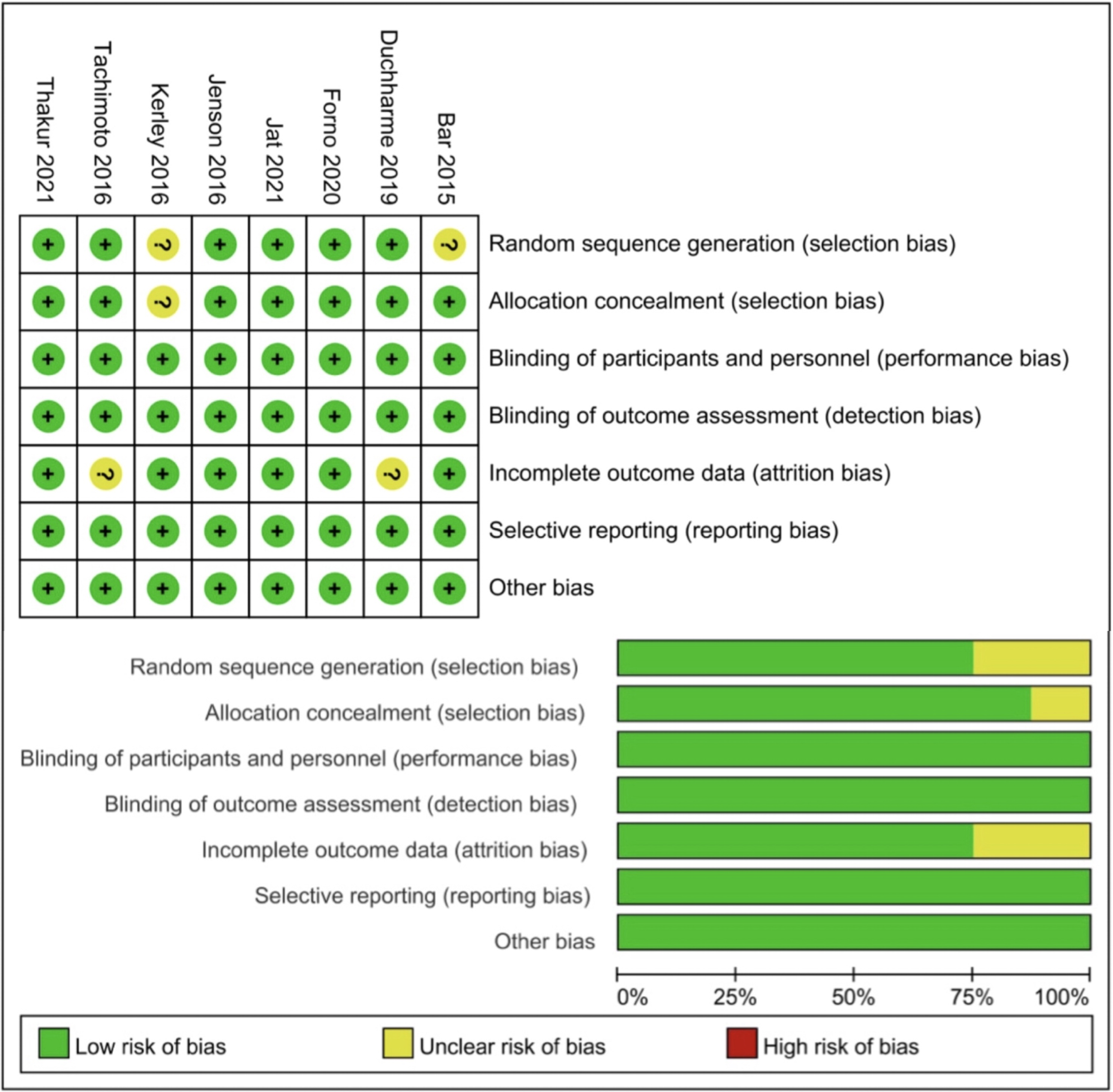

Supplement: Supplementary Figure S1 — Risk of bias graph for included studies. [file Image_1.TIF]

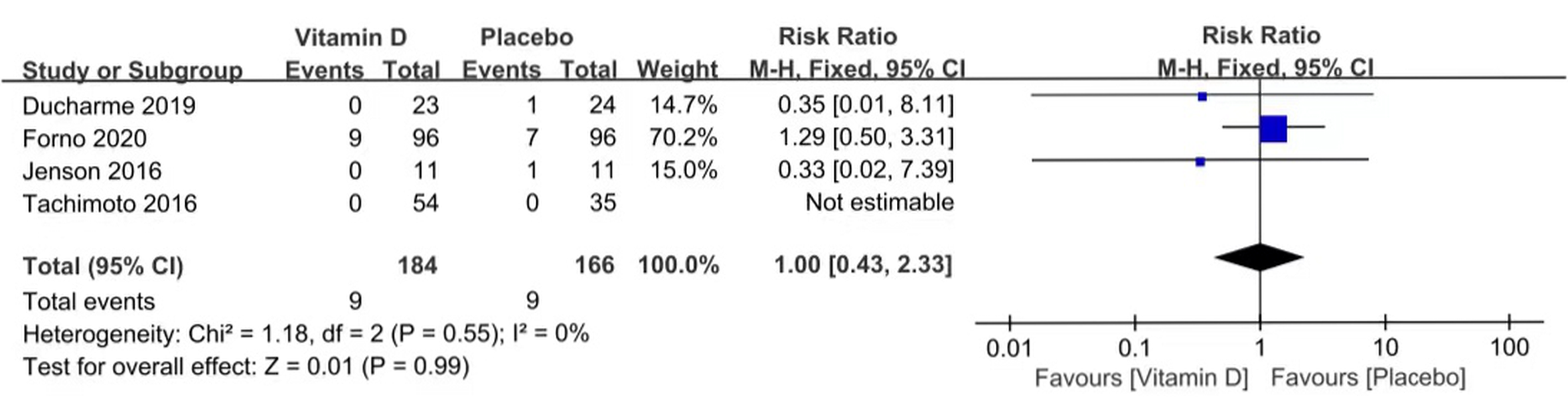

Supplement: Supplementary Figure S2 — Forest plots RR of serious adverse events associated with vitamin D versus placebo. [file Image_2.TIF]

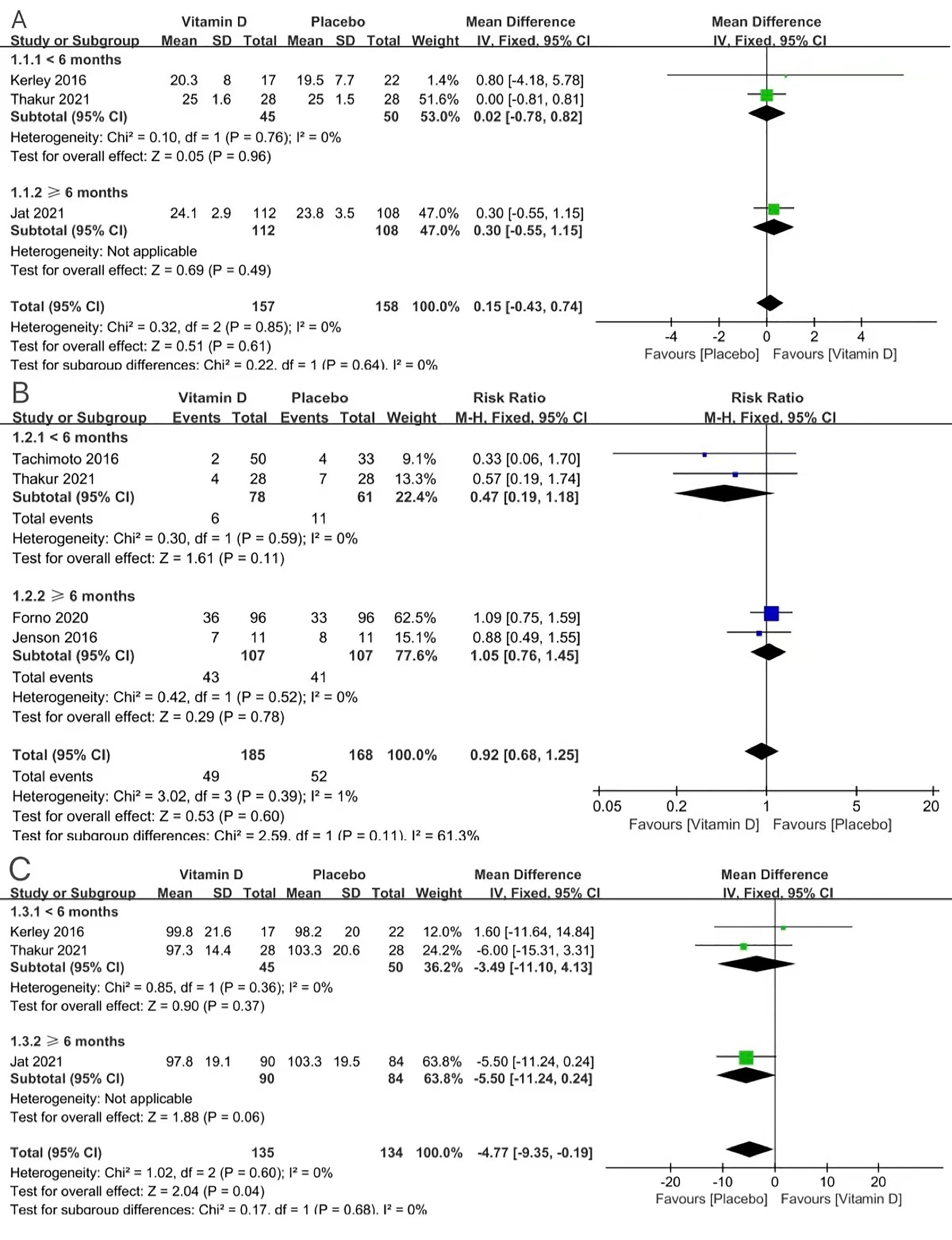

Supplement: Supplementary Figure S3 — Subgroup analysis of CACT scores, asthma exacerbation and FEV1%. [file Image_3.JPEG]

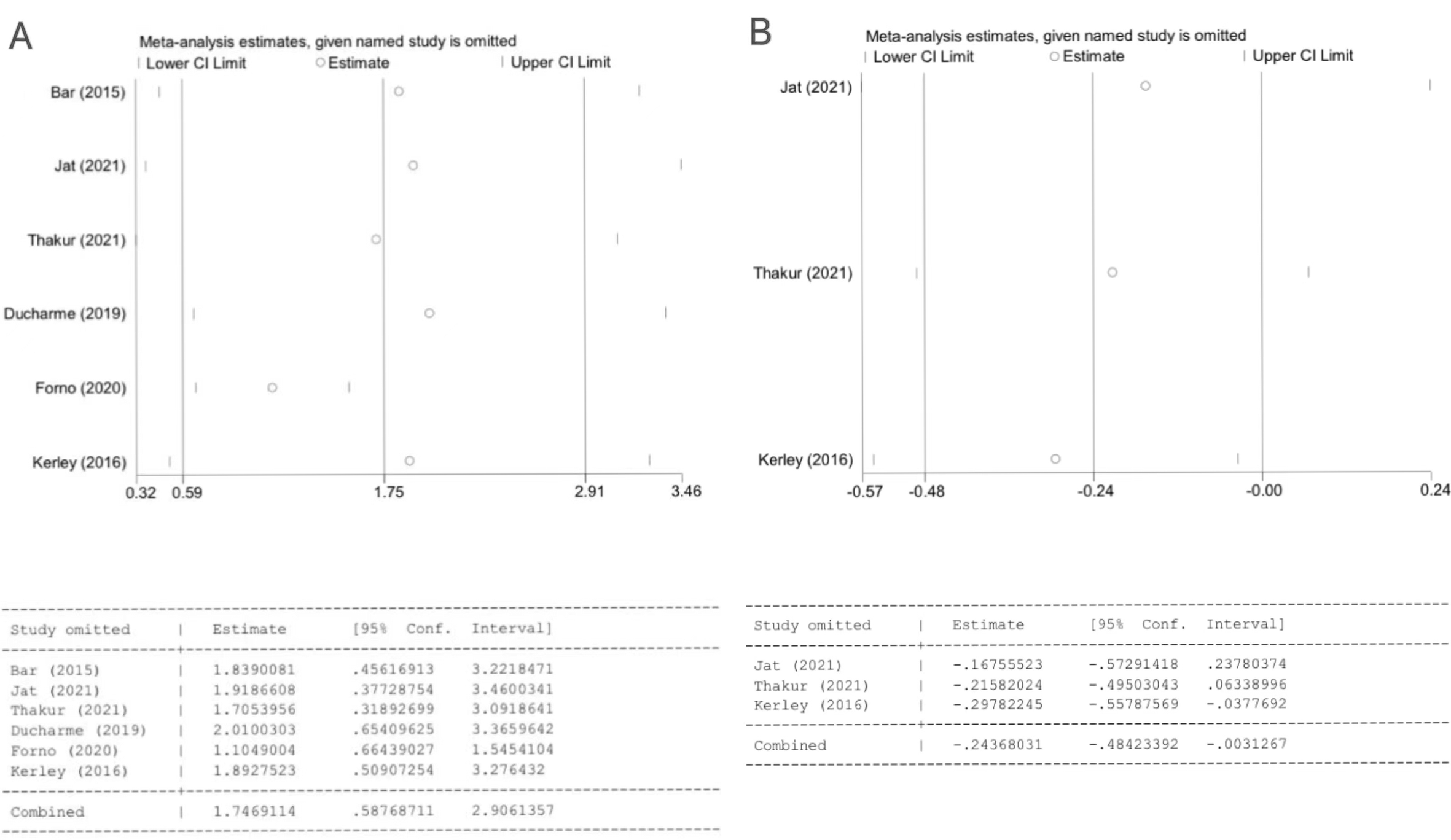

Supplement: Supplementary Figure S4 — Meta-based influence analyses for vitamin D levels (A) and FEV1% (B). [file Image_4.TIF]

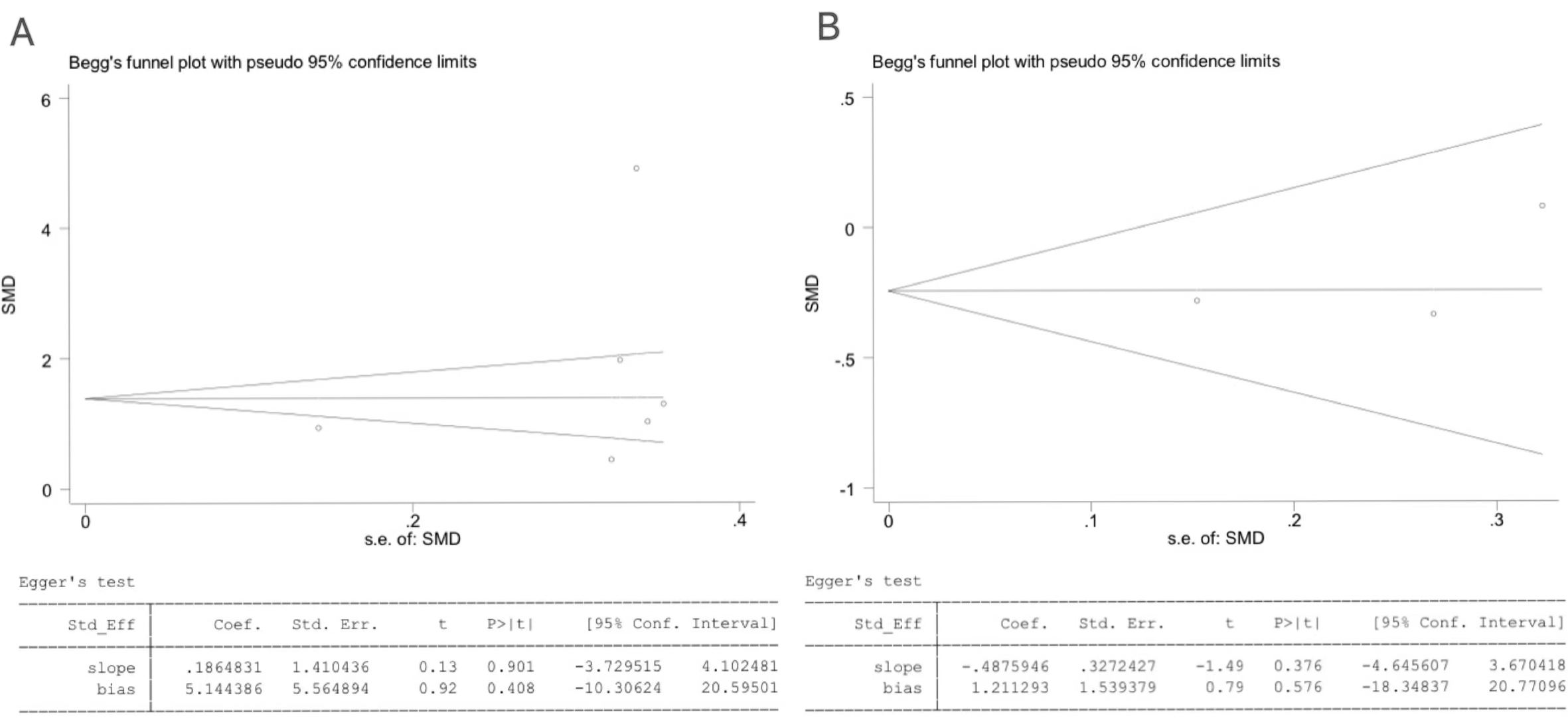

Supplement: Supplementary Figure S5 — Egger's and Begg's tests for comparisons of vitamin D levels (A) and FEV1% (B). [file Image_5.TIF]
