# Supplementary material for: The Effect of Vitamin D Supplementation in Children With Asthma: A Meta-Analysis
Source: Front Pediatr. 2022 Jun 29;10:840617. doi: 10.3389/fped.2022.840617 (PMC9277022; doi:10.3389/fped.2022.840617)
Supplement: Supplementary file 7 [file Table_2.DOCX]

**Table S2** Search strategy.

| **PubMed**  The database was searched on October 24, 2021, n=112.  Search Strategy:  (Vitamin D[Title/Abstract] OR 25-hydroxyvitamin D(25(OH)D)[Title/Abstract] OR Vitamin D-3[Title/Abstract] OR 25-hydroxyvitamin D[Title/Abstract] OR Cholecalciferol[Title/Abstract]) AND ( Children [Title/Abstract]) AND (Asthma[Title/Abstract] OR Asthmas[Title/Abstract] OR Bronchial Asthma[Title/Abstract] OR Asthma, Bronchial[Title/Abstract]) |
| --- |
| **Web of Science**  The database was searched on October 24, 2021, n=935.  Search Strategy:  1 TOPIC: (“Vitamin D” OR “25-hydroxyvitamin D (25(OH)D)” OR “Vitamin D-3” OR “25-hydroxyvitamin D” OR “Cholecalciferol”) (79474)  2 TOPIC: (“Children”) ( 1,365,804)  3 TOPIC: (“Asthma” OR “Asthmas” OR “Bronchial Asthma” OR “Asthma, Bronchial”) ( 141,417)  4 #1 AND #2 AND #3 (935) |
| **EMBASE**  The database was searched on October 24, 2021, n=759.  Search Strategy:  ('Children':ti,ab,kw) AND ('Vitamin D':ti,ab,kw OR '25-hydroxyvitamin D (25(OH)D)':ti,ab,kw OR 'Vitamin D-3':ti,ab,kw OR '25-hydroxyvitamin D':ti,ab,kw OR 'Cholecalciferol':ti,ab,kw) AND ('Asthma':ti,ab,kw OR 'Asthmas':ti,ab,kw OR 'Bronchial Asthma':ti,ab,kw OR 'Asthma, Bronchial':ti,ab,kw) |
| **Cochrane Library**  The database was searched on October 24, 2021, n=186.  Search Strategy:  (“Children”): ti,ab,kw AND (“Vitamin D” OR “25-OH-D” OR “Vitamin D-3” OR “25-hydroxyvitamin-D” OR “Cholecalciferol”): ti,ab,kw AND (“Asthma” OR “Asthmas” OR “Bronchial Asthma” OR “Asthma, Bronchial”): ti,ab,kw - (Word variations have been searched) |
| **Ovid MEDLINE**  The database was searched on October 24, 2021, n=1443.  Search Strategy:  1 Children.ab. (3418808)  2 Vitamin D.ab. (196417)  3 25-OH-D.ab. (36041)  4 Vitamin D-3.ab. (2769)  5 25-hydroxyvitamin D.ab. (40034)  6 Cholecalciferol .ab. (9479)  7 or/2-6 [ Vitamin D ].ab. (207328)  8 Asthma .ab. (426887)  9 Asthmas .ab. (385)  10 Bronchial Asthma .ab. (32060)  11 Asthma, Bronchial .ab. (860)  12 or/8-11 [ Asthma ] (426919)  13 1 and 7 and 12 (1443) |
| **ScienceDirect**  The database was searched on October 24, 2021, n=251.  Search Strategy:  Title, abstract, keywords: ((“Children”) and (“Vitamin D” OR “25-hydroxyvitamin D (25(OH)D” OR “Vitamin D-3” OR “25-hydroxyvitamin D” OR “Cholecalciferol”) and (“Asthma” OR “Asthmas” OR “Bronchial Asthma” OR “Asthma, Bronchial”)) |
| **Scopus**  The database was searched on October 24, 2021, n=891.  Search Strategy:  TITLE-ABS-KEY ((“Children”) and (“Vitamin D” OR “25-hydroxyvitamin D (25(OH)D” OR “Vitamin D-3” OR “25-hydroxyvitamin D” OR “Cholecalciferol”) and (“Asthma” OR “Asthmas” OR “Bronchial Asthma” OR “Asthma, Bronchial”)) |

**Note:** The combined text and medical subject heading (MeSH) terms used were: “Asthma”, “Children” and “Vitamin D”.
