# Supplementary material for: The Effect of Vitamin D Supplementation in Children With Asthma: A Meta-Analysis
Source: Front Pediatr. 2022 Jun 29;10:840617. doi: 10.3389/fped.2022.840617 (PMC9277022; doi:10.3389/fped.2022.840617)
Supplement: Supplementary file 8 [file Table_3.DOCX]

**Table S3** Quality assessment of all included studies

| **Study** | | **Randomization** | **Masking** | **Accountability of all patients** | **Quality (score)** |
| --- | --- | --- | --- | --- | --- |
| 2021 | Thakur [25] | ★★ | ★★ | ★ | 5 |
| 2021 | Jat [17] | ★★ | ★★ | ★ | 5 |
| 2020 | Forno [24] | ★★ | ★★ | ★ | 5 |
| 2019 | Ducharme [23] | ★★ | ★★ | ★ | 5 |
| 2016 | Tachimoto [21] | ★★ | ★★ | ★ | 5 |
| 2016 | Keyley [16] | ★ | ★ | ★ | 3 |
| 2016 | Jenson [22] | ★★ | ★★ | ★ | 5 |
| 2015 | Bar [20] | ★★ | ★★ | ★ | 4 |
