# Supplementary material for: The Effect of Vitamin D Supplementation in Children With Asthma: A Meta-Analysis
Source: Front Pediatr. 2022 Jun 29;10:840617. doi: 10.3389/fped.2022.840617 (PMC9277022; doi:10.3389/fped.2022.840617)
Supplement: Supplementary file 9 [file Table_4.DOCX]

**Table S4** GRADE Quality assessment by therapeutic strategy and study design for the outcomes of asthma control, lung function, safety and vitamin D levels.

| **Primary outcomes** | **No. of Studies** | **No. of participants** | | **Differences^a^（95%CI）** | **Quality assessment** | | | | | **Quality** |
| --- | --- | --- | --- | --- | --- | --- | --- | --- | --- | --- |
|  |  | **Vitamin D** | **Placebo** |  | **Risk of bias^b^** | **Inconsistency** | **Indirectness** | **Imprecision** | **Publication bias^c^** |  |
| **Vitamin D levels** |  |  |  |  |  |  |  |  |  |  |
| Baseline | 8 | 375 | 362 | 0.14 [-1.13, 1.42] | Serious (-1) | Serious (-1) | No indirectness | Serious (-1) | Unlikely | Very Low |
| After intervention | 6 | 262 | 272 | 13.51 [4.24, 22.79] | Serious (-1) | Very Serious (-2) | No indirectness | Serious (-1) | Unlikely | Very Low |
| **Asthma control** |  |  |  |  |  |  |  |  |  |  |
| CACT score |  |  |  |  |  |  |  |  |  |  |
| Baseline | 5 | 322 | 308 | 0.29 [-0.21, 0.79] | Serious (-1) | Very Serious (-2) | No indirectness | No imprecision | Unlikely | Very Low |
| After intervention | 3 | 157 | 158 | 0.15 [-0.43, 0.74] | Serious (-1) | No inconsistency | No indirectness | Serious (-1) | Unlikely | Low |
| Asthma exacerbation | 5 | 310 | 294 | 0.92 [0.68, 1.25] | Serious (-1) | No inconsistency | No indirectness | Serious (-1) | Unlikely | Low |
| Hospitalizations for asthma exacerbation | 4 | 189 | 170 | 1.20 [0.48, 2.96] | Serious (-1) | No inconsistency | No indirectness | Very Serious (-2) | Unlikely | Very Low |
| Acute care visits | 3 | 62 | 63 | 1.13 [0.77, 1.65] | Serious (-1) | No inconsistency | No indirectness | Very Serious (-2) | Unlikely | Very Low |
| Steriod use | 4 | 122 | 109 | 1.03 [0.41, 2.57] | Serious (-1) | Serious (-1) | No indirectness | Serious (-1) | Unlikely | Very Low |
| FeNO |  |  |  |  |  |  |  |  |  |  |
| Baseline | 2 | 50 | 49 | -4.78 [-14.64, 5.07] | Serious (-1) | No inconsistency | No indirectness | Serious (-1) | Unlikely | Low |
| After intervention | 2 | 48 | 47 | 1.98 [-3.54, 7.50] | Serious (-1) | Serious (-1) | No indirectness | Serious (-1) | Unlikely | Very Low |
| **Lung function** |  |  |  |  |  |  |  |  |  |  |
| FEV1% |  |  |  |  |  |  |  |  |  |  |
| Baseline | 5 | 322 | 308 | 0.18 [-1.72, 2.08] | Serious (-1) | No inconsistency | No indirectness | Serious (-1) | Unlikely | Low |
| After intervention | 3 | 135 | 134 | -4.77 [-9.35, -0.19] | Serious (-1) | No inconsistency | No indirectness | Serious (-1) | Unlikely | Low |
| FVC% |  |  |  |  |  |  |  |  |  |  |
| Baseline | 3 | 196 | 182 | 0.35 [-2.71, 3.41] | Serious (-1) | No inconsistency | No indirectness | Serious (-1) | Unlikely | Low |
| After intervention | 2 | 107 | 106 | -4.67 [-9.52, 0.18] | Serious (-1) | No inconsistency | No indirectness | Serious (-1) | Unlikely | Low |
| FEV1:FVC |  |  |  |  |  |  |  |  |  |  |
| Baseline | 4 | 292 | 278 | 0.73 [-0.75, 2.21] | Serious (-1) | No inconsistency | No indirectness | Very Serious (-2) | Unlikely | Very Low |
| After intervention | 2 | 107 | 106 | -0.86 [-3.52, 1.79] | Serious (-1) | No inconsistency | No indirectness | Very Serious (-2) | Unlikely | Very Low |
| **Safety** |  |  |  |  |  |  |  |  |  |  |
| Serious adverse events | 4 | 184 | 166 | 1.00 [0.43, 2.33] | Serious (-1) | No inconsistency | No indirectness | Very Serious (-2) | Unlikely | Very Low |
| Hypercalciuria | 3 | 62 | 63 | 0.58 [0.22, 1.53] | Serious (-1) | No inconsistency | No indirectness | Serious (-1) | Unlikely | Low |
| Hospital admission | 4 | 160 | 161 | 0.84 [0.37, 1.87] | Serious (-1) | No inconsistency | No indirectness | Very Serious (-2) | Unlikely | Very Low |

**Abbreviations:** CACT: childhood  asthma control test; FEV1% :  predicted percentage of forced expiratory volume in first second; FVC%: percentage of predicted forced vital capacity; CI: confidence interval.

^a^ Differences: mean difference (MD) for CACT scores, FeNO, lung function and vitamin D levels; odds ratios (OR) for safety, asthma exacerbation, hospitalizations for asthma exacerbation, acute care visits and steriod use.

^b^ Blinding method and selective reporting and other types of some included trials were not offered.

^c^ Publication bias were reported by incomplete outcome data.
